# Small Bowel Obstruction Caused by an Incarcerated Hernia after Iliac Crest Bone Harvest

**DOI:** 10.5402/2011/836568

**Published:** 2011-04-14

**Authors:** Steven d'Hondt, Savas Soysal, Philipp Kirchhoff, Daniel Oertli, Oleg Heizmann

**Affiliations:** Department of Visceral Surgery, University of Basel, 4031 Basel, Switzerland

## Abstract

The iliac crest has become an often used site for autogenous bone graft, because of the easy access it affords. One of the less common complications that can occur after removal is a graft-site hernia. It was first reported in 1945 (see the work by Oldfield, 1945). We report a case of iliac crest bone hernia in a 53-year-old male who was admitted for elective resection of a pseudarthrosis and reconstruction of the left femur with iliac crest bone from the right side. One and a half months after initial surgery, the patient presented with increasing abdominal pain and signs of bowel obstruction. A CT scan of the abdominal cavity showed an obstruction of the small bowel caused by the bone defect of the right iliac crest. A laparoscopy showed a herniation of the small bowel. Due to collateral vessels of the peritoneum caused by portal hypertension, an IPOM (intraperitoneal onlay-mesh) occlusion could not be performed. We performed a conventional ventral hernia repair with an onlay mesh. The recovery was uneventful.

## 1. Background

Main causes of bowel obstructions are postoperative adhesions, second leading causes are malignant tumors or strictures of the small bowel, followed by hernias [1]. Intussusception, volvulus, Crohn's disease, and gallstones (gallstone ileus) account for only a small percentage of cases. Since the iliac crest has become an often used site for autogenous bone graft, one of the rare complications is a graft site hernia. 

## 2. Case Presentation

A 53-year-old man was admitted for the elective resection of a pseudarthrosis and reconstruction of the left femur with iliac crest bone from the right side. The patient also suffered from liver cirrhosis Child B and thrombosis of the portal vein due to infection with hepatitis C and alcohol abuse. A hepatocellular carcinoma was diagnosed in a liver biopsy performed during hospitalisation.

At the right iliac crest bone harvest point, two fragments of artificial bone, which had been inserted for stabilisation, subsequently dislocated. Because of the patient's limited health, there was no immediate removal of the dislocated fragments. 

**Figure 1:**
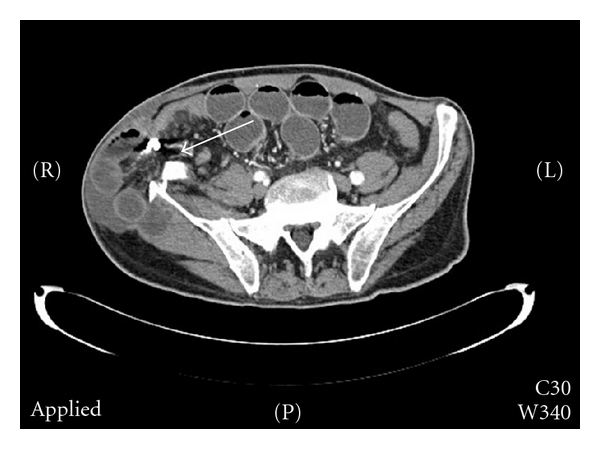
Arrow pointing to the herniated bowel and tutoplast cube.

In the course of further hospitalisation, the patient complained of increasing abdominal pain and showed signs of bowel obstruction. A lump on the right iliac crest could be palpated, and bowel movement was auscultated. A CT of the abdomen confirmed an obstruction of the small bowel caused by the herniation of the ileum into the the bone defect in the right iliac crest (Figure 1). 

**Figure 2:**
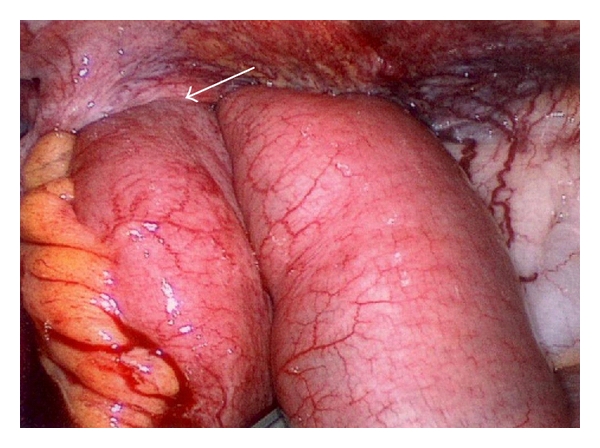
Hernia before repositioning of small bowel.

**Figure 3:**
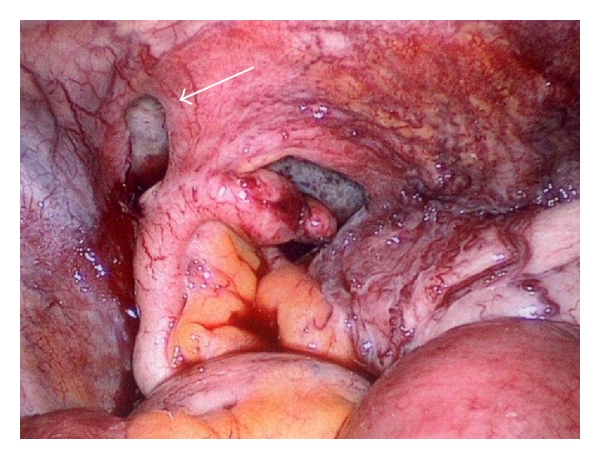
Hernia after repositioning.

During laparoscopy, no sign of ischemia in the herniated part of the bowel could be seen. The small bowel had herniated directly below the cecal pole through the peritoneum (Figures 2, and 3). Due to collateral circulation in the peritoneum and cecum caused by portal hypertension, an intraperitoneal onlay-mesh repair could not be performed. After the trocars were removed, the wound at the right iliac crest was reopened. Then, a DynaMesh of 12 × 13 cm was fixed with single sutures. After returning to a normal diet and an uneventful recovery, the patient was transferred to an orthopedic rehabilitation clinic for further recovery.

## 3. Discussion

Hernia symptoms range from a palpable lump in the groin or abdomen, pain, and tenderness, to nausea and vomiting when obstruction occurs. In most cases, the hernia should be corrected surgically to avoid incarceration, strangulation or intestinal obstruction.

Since the iliac crest has become a common donor site for autogenous bone graft, numbers of postsurgical inferior lumbar hernias are rising. The fist case was reported in 1945 [2]. The incidence varies between 5–9% as shown in previous reports [3, 4]. Other reported complications are nerve and ureteral injury, arterial injury, fracture, pelvic instability, herniation, ileus, and hematoma [5]. Due to the increased morbidity and mortality associated with these defects, the treatment of choice is surgical repair. Hernia incarceration occurs in 25% and strangulation in 10% of the cases [6]. Tension-free mesh repair is nowadays most frequently performed. It can be accomplished through laparoscopic, retroperitoneal or transabdominal approach. The first laparoscopic repair of lumbar hernia was described in 1996 by Burick and Parascandola [7]. Other techniques include soft tissue transfers to bridge the gap of the abdominal wall defect (transversalis fascia, abdominal musculature, and tensor fascia lata) or the Bosworth repair (the anterior superior iliac spine is moved inferiorly and posteriorly, so muscles and fascia cover the defect). A new corkscrew anchor system has been described by Patten et al. to place sutures in the postharvest iliac crest remnant [8]. As described, we repaired the extraperitoneal hernia after iliac crest bone harvest with an onlay mesh.

## 4. Conclusion

If symptoms of bowel obstruction occur after an iliac bone crest harvest, it is important to think of a hernia. While removing the iliac bone crest, verification of an intact peritoneum is important. A CT scan confirms the diagnosis and is useful to plan the surgical repair. A laparoscopic repair should be performed if possible, as it has proved to be safe for the repair of hernias through the inferior lumbar triangle [9, 10].
